# Comparing Hydrolysable and Condensed Tannins for Tannin Protein-Based Foams

**DOI:** 10.3390/polym17020153

**Published:** 2025-01-09

**Authors:** Jonas Eckardt, Lorenzo Moro, Elena Colusso, Primož Šket, Samuele Giovando, Gianluca Tondi

**Affiliations:** 1 Department of Land, Environment, Agriculture and Forestry, University of Padua, Viale dell’Università 16, 35020 Padua, Italy; jonasraphael.eckardt@phd.unipd.it; 2 Department of Industrial Engineering, University of Padova, Via Marzolo 9, 35131 Padua, Italy; lorenzo.moro@unipd.it (L.M.); elena.colusso@unipd.it (E.C.); 3 Slovenian NMR Centre, National Institute of Chemistry, Hajdrihova 19, 1000 Ljubljana, Slovenia; primoz.sket@ki.si; 4 Centro Ricerche per la Chimica Fine Srl for Silvateam Spa, Via Torre 7, 12080 San Michele Mondovì, Italy; sgiovando@silvateam.com

**Keywords:** bio-based, flavonoid, castalagin, soy protein isolate, hexamine, ^13^C NMR

## Abstract

Tannin-based foams have gained attention as a potential bio-based alternative to conventional synthetic foams. Traditionally, namely condensed tannins (CT) have been used, leaving the potential of hydrolysable tannins (HT) largely unexplored. This study compared the performance of chestnut (HT) and quebracho (CT) in tannin–protein-based foams at different tannin ratios. Using soy protein isolate (SPI) and hexamine under acidic conditions, a series of tannin foams were produced through a mechanical foaming method and analyzed for cell structure, compression strength, thermal conductivity, and chemical stability. Results show that chestnut tannin is viable in hexamine SPI formulations but is harder to process due to lower reactivity, further resulting in higher material densities compared to quebracho. Foams with higher quebracho content featured smaller, more interconnected cells, while increasing chestnut content led to larger, less interconnected cells. Compression strength decreased with higher chestnut content, while fire resistance and thermal conductivity were influenced by material density rather than tannin type. The ^13^C-NMR analysis revealed covalent bonding of hexamine with both tannins, but potential covalent bonds with SPI were undetectable. Overall, chestnut tannin can substitute quebracho tannin in hexamine-SPI foams, though with compromises in terms of specific material properties and processability.

## 1. Introduction

The increasing interest in sustainable materials has driven significant research into bio-based foams as alternatives to conventional synthetic foams such as polyurethane or XPS [1,2,3]. Among these, tannin-based foams have attracted attention for their potential to produce highly porous and lightweight materials with good thermal insulating and fire-resistant properties [3,4,5]. These foams can also be functionalized, enabling them to become, e.g., semi-conductive [6] or hydrophobic [7], and can further be carbonized for additional functionality [8,9]. Moreover, these foams can mitigate ammonia emissions from cattle slurry [10] and absorb pollutants, including heavy metals and cationic dyes [11,12]. Tannins, a class of polyphenolic compounds derived from different plant sources, can chemically be categorized into different groups such as hydrolysable tannins (HT) and condensed tannins (CT) [13,14]. While CTs have been extensively researched for their use in tannin-based adhesives [15], foams [4], and other polymeric materials [16,17], few studies have recently begun to investigate the potential of HTs for foam production [18,19,20]. These investigations have shown that HT can also be effectively utilized in foam production by using furfuryl alcohol [18] as a crosslinker or employing non-isocyanate polyurethane (NIPU) chemistry [20]. However, despite their potential, these tannins present processing challenges due to their lower chemical reactivity, which resulted in furanic foams with reduced mechanical strength and increased material density compared to foams made from CT [18]. Since it is well-known that different crosslinking systems yield varying material properties in CT foams [4,21], it is logical to explore similar options for HT foams.

Initial research into tannin foams primarily utilized formaldehyde as a crosslinker [22], but over the years, the focus has shifted to alternatives such as furfuryl alcohol [23,24], hexamine [25,26], and polyurethane-based systems [27,28]. Additionally, tannins have a long history of use as tanning agents due to their ability to chemically interact with proteins [29]. Proteins from both plant and animal sources have been successfully incorporated into tannin foam production, leveraging their natural surfactant properties and ability to engage in chemical reactions [30,31]. These interactions are primarily driven by the phenolic hydroxyl groups in tannins, which can form ionic, hydrogen, and covalent bonds with the cationic amino groups of proteins, while hydrogen bonds can also form between the carbonyl groups in the protein peptide linkage of the protein and the hydroxyl groups in tannins [31,32,33,34].

In the context of CT furanic foams, incorporating soy protein isolate (SPI) has been shown to enhance mechanical properties, reduce pulverization, and improve thermal stability [31]. To further optimize these properties, hexamine is often used as a co-crosslinker in tannin–protein systems. The addition of hexamine can increase the crosslinking density within the polymer network, resulting in improved mechanical strength, thermal stability, and water resistance [33].

While CTs have demonstrated good usability in foam production, there remains a significant knowledge gap about the potential of HT, especially when employing a protein-supported crosslinking approach. To address this gap, the presented study explores the use of HTs in a tannin–protein-based foam formulation. Building on previous research that utilized albumin as a protein [30] and guided by various tannin–SPI adhesive and foam formulations [32,33,35,36], we developed a mechanically foamed formulation using hexamine and SPI as crosslinkers under acidic conditions. Different ratios of CT from quebracho and HT from chestnut were tested to understand the differences between these two types of tannins and to gather insights on the potential usability of mixed tannin extracts. The resulting foams were then analyzed for their cell structure, compression and fire resistance, thermal conductivity, and stability against acids, bases, and non-polar solvents. Additionally, a ^13^C-NMR analysis was conducted to further explore the polymeric chemistry involved. Through this work, we aim to expand the knowledge base on HT foam production and to explore additional bio-based building blocks for foamed polymers.

## 2. Materials and Methods

### 2.1. Chemicals and Reagents

Industrial tannin extracts of Chestnut (*Castanea sativa*) and Quebracho (*Schinopsis balancae*) were supplied by Silvateam S.p.A. (S. Michele Mondovì, Italy). Soy protein isolate, with a protein content greater than 90%, was purchased from Special Ingredients Ltd. (Chesterfield, UK). Glycerol, ethylene glycol, and hexamine were ordered from Thermo Fisher Scientific (Waltham, MA, USA). Tween80 and sulfuric acid were obtained from Merck (Darmstadt, Germany). Before usage, hexamine was diluted to 33 wt% and sulfuric acid to 32 wt%.

### 2.2. Foam Synthesis

Foams were produced through mechanical agitation using a VELP (Usmate Velate, Italy) overhead stirrer, equipped with a butterfly stirring head from VMA-Getzmann GmBH (Reichshof, Germany).

Initially, tannin was dissolved in a beaker containing a mixture of ethylene glycol, glycerol, and water under mechanical agitation at 800 rpm. Hexamine was then gradually introduced, followed by the addition of sulfuric acid. In the final step, SPI and Tween 80 were added, and the stirring speed was increased to 1200 rpm. After 20 min, a stable wet foam mass was formed. This mixture was then transferred into a PTFE-coated mold and cured in a convection oven at 80 °C for 24 h. Samples for the fire test, compression test, and cell analysis were produced in a mold measuring 10 × 10 × 4 cm^3^, while samples for the thermal conductivity test were produced in a mold measuring 15 × 15 × 7 cm^3^. The weight percentages of the different constituents used in the formulations are detailed in Table 1, of which hexamine and sulfuric acid were additionally diluted to 33 wt% and 32 wt%, respectively.

The tannin component was adjusted to five different chestnut and quebracho tannin ratios by weight: 0:100, 15:85, 50:50, 85:15, and 100:0. The acronym 0, 15, 50, 85, and 100 therefore refers to the amount of chestnut tannin used in the formulation.

### 2.3. Density and Porosity

To determine the bulk density (d), also defined as geometrical density, the outside layer (0.5 cm) of each foam sample was removed, and the remaining core was cut into rectangular blocks. The density (ρ) was then calculated by measuring the sample’s length (l), width (w), height (h), and weight (m), using the formula d = m/(l × w × h). The apparent (da) and skeletal density (ds) of the foams were measured using a He-pycnometer (Anton Paar (Graz, Austria), Ultrapyc 3000). Samples were dried at 60 °C for 4 h before the test. The total porosity of the foams was calculated according to equation $P=(1-\frac{d}{ds})\times 100$, while the open porosity was calculated according to $Po=(1-\frac{d}{da})\times 100$, allowing the open pore percentage to be determined as (Po/P) × 100.

### 2.4. Surface Area

The specific surface area was estimated by nitrogen adsorption at 77 K using an automated gas adsorption analyzer (Autosorb iQ Anton Paar, Graz, Austria). Samples were degassed at 120 °C for 18 h before measurements. The surface area was calculated based on the Brunauer, Emmett, Teller (BET) isotherms in the relative pressure range of 0.075–0.23.

### 2.5. Cell Measurements

Cell size dimensions were measured using the stereo microscope LEICA M80 (Wetzlar, Germany) equipped with a LEICA Camera model DMC4500 and by using the image acquisition software LAS version 4.13. For each formulation, at least 50 length measurements and 100 width measurements were taken parallel to the growth direction. Based on a method already applied for tannin foams [37,38], the average cell diameter (Cell Ø) was then calculated using the formula: Cell Ø = (π/4) × D, where D represents the mean of 150 measurements. Orthotropicity was calculated by dividing the length of each cell by its corresponding width. Pictures of at least five different samples were analyzed. To maintain comparability, extremely large and small pores were excluded from the measurements.

### 2.6. Scanning Electron Microscopy (SEM)

Morphological characterizations of the samples were performed with an ESEM Quanta 200 (FEI, Hillsboro, OR, USA), equipped with a backscattered electron detector, operating at 20 Kv in low vacuum conditions. A representative sample (1 cm^3^) was cut from the foams and fixed on the sample holder with carbon tape. Samples did not receive any treatment before the analysis.

### 2.7. Solid State ^13^C NMR

The ^13^C-CP-MAS NMR spectra of leached foam samples, including those with 0% and 100% Chestnut tannin as well as various combinations of formulation components, were recorded using a Bruker Avance NEO 400 MHz NMR spectrometer (Bruker BioSpin, Rheinstetten, Germany) equipped with a 4 mm CP-MAS probe. The samples were spun at a frequency of 10 kHz at 25 °C. A total of 13,000 scans were accumulated with the repetition delay of 3 s.

### 2.8. Compression Test

Compression strength was determined using a Galdabini Quasar 25 (Galdabini, Cardano-Varese, Italy). Cubes of 3 × 3 × 3 cm^3^ were tested parallel to the growth direction at a testing rate of 2 mm/min. If a distinct break in the material was observed within 10% deformation of the specimen’s height, that value was recorded as the maximum compression strength. If no clear break occurred within this range, the compression strength value at 10% deformation was used for comparison. For each formulation, 10 foam cubes were tested.

### 2.9. Thermal Conductivity

The thermal conductivity was measured using the Transient Plane Source (TPS) technique with the Hot Disk 3500 TPS apparatus (Hot Disk AB, Gothenburg, Sweden). The device uses a double-spiral nickel foil sensor, insulated with two Kapton layers, that is placed between two foam samples of a similar formulation, each measuring 4.5 × 4.5 × 2.5 cm^3^. For these measurements, sensor 8563 (radius 9.863 mm) was selected, with power set to 60 mW and a measurement time of 320 s. The sensor works as a resistance thermometer, enabling the measurement of temperature increase over time on the active surfaces of the specimens due to the current supplied. A detailed description of this technique has been reported in several studies [39,40], allowing the thermal conductivity of the tested foams to be calculated in W/(m·K). The measurements were taken under dry conditions at room temperature (~293 ± 2 K), and the reported values represent the average of at least five measurements.

### 2.10. Leaching Resistance and Acid Recovery

The leaching resistance of the different foams was evaluated with solvents at different polarities, such as water, ethanol, and hexane, as well as in an alkaline (NaOH, pH 13) and acidic solution (H_2_SO_4_, pH 2). The testing procedure involved crushing the foams and drying them at 103 °C. A 4 g sample of the foam powder was then added to 100 mL of the solvent and stirred for 2 h. Afterward, the mixture was filtered using a paper filter with a pore size of 25 µm. The filtered powder was dried and weighed to determine the percentage of material leached, based on the weight difference. This process was repeated five times for each foam formulation and leaching test.

### 2.11. Fire Test

Fire resistance was tested based on a method previously used by the group [38]. Foam cubes with dimensions of 2.5 cm per side were placed on a metal grid positioned on a laboratory tripod, which was set on an analytical balance. The samples were then directly exposed to the oxidant flame of a Bunsen burner for 3 min, maintaining a distance of 5 cm from the burner’s opening. The weight was recorded every 15 s during the exposure period and once more when no further weight loss was observed. Five samples from each formulation were tested, and the average percentage of mass loss was calculated for each formulation.

### 2.12. Statistical Analysis and Data Processing

All statistical analyses were conducted using SPSS Version 27 (IBM, Armonk, NY, USA). Several multiple regression models were evaluated, in which tannin type and other measured material properties served as predictors. The significance level was set at *p* < 0.05.

## 3. Results and Discussion

Before detailing the characterization of the produced foams, it is important to highlight several findings from the initial pretrials, which clarify the rationale behind the development of these formulations. Initial pretrials with hexamine alone resulted in very brittle Quebracho foams and failed to produce chestnut foams due to the low reactivity of chestnut tannin, causing the foam to collapse before hardening. Adding SPI addressed this issue by extending the stability of the wet foam mass until hardening of the material could occur. Scaling up the process without adjusting production parameters resulted in different material densities for a chestnut content of 50% and higher in relation to quebracho tannin. Particularly, producing an upscaled chestnut-only foam resulted in very different material as it exhibited partial cell collapse. This is likely due to changes in the steering equipment-to-liquid ratio, along with variations in heat distribution and vapor evaporation, which poses a significant challenge during the upscaling of the chestnut foams.

### 3.1. A-Morphology

**Figure 1:**
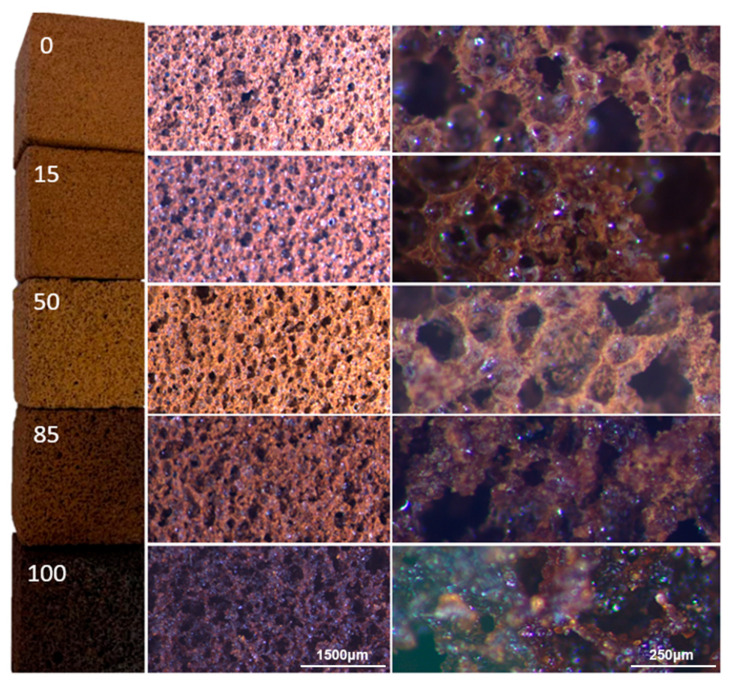
Different foams with magnified views of cell structure. (**Left**): macroscopic view; center: optical microscope at 10×; (**right**): optical microscope at 60× magnification.

The resulting foams of the five foam formulations presented in this work are shown in Figure 1.

**Figure 2:**
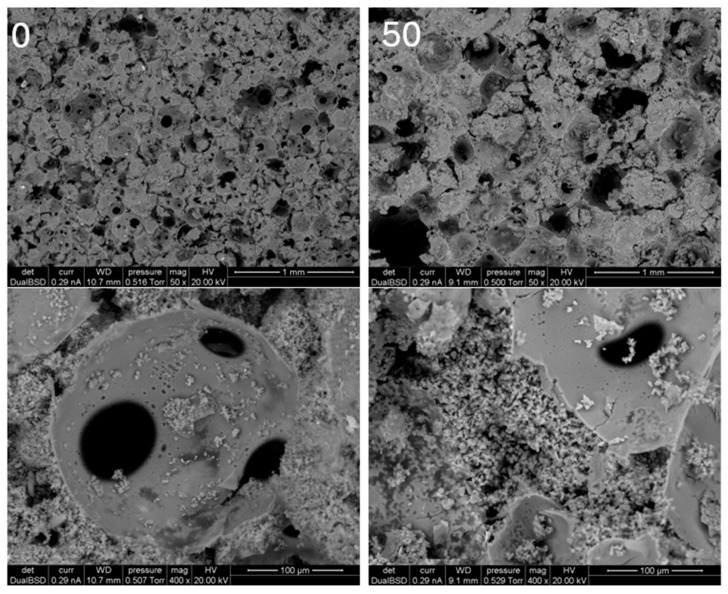
Electron microscope pictures of foams with 50 (**right**) and 0 percent (**left**) chestnut at 50× (**top**) and 400× (**bottom**) magnification.

The foams with a low chestnut tannin content appear brown, homogeneous, and highly porous, featuring well-interconnected cells. As the amount of chestnut tannin increases, the color becomes darker, and also the pore structure becomes rougher. The cell walls of foams with 85% and 100% chestnut tannin appear more agglomerated, with larger cells and fewer very small cells compared to those with lower chestnut tannin content. This trend becomes more evident in the SEM images in Figure 2, where the 50% chestnut foam shows thicker walls, fewer interconnections between cells, and a sparse distribution of small cells.

Additionally, small agglomerates of Tween are observed around the cells, consistent with literature findings [41]. The results of measuring the cell size and orthotropicity are displayed in Table 2.

As the amount of chestnut tannin increases, cell size and density also rise, along with a slight increase in orthotropicity. Contrary to expectations, density decreases as cell size decreases, likely due to thinner cell walls, the presence of micropores, and increased interconnections. The foams display slightly higher orthotropicity than mechanically foamed furanic foams, likely due to the lower reactivity of the crosslinking system compared to furfuryl alcohol, which may lead to a slight collapse of the cell structure [18]. Compared to tannin–furanic foams produced with a blowing agent, they tend to have lower orthotropicity [37,38]. Helium pycnometry measurements were also conducted for the foam with 0% and 50% chestnut tannin, and the results have highlighted similar skeletal density of 1.45 g/cm^3^. This measurement allowed us to calculate the total porosities, which were higher than 80% (84.4% for 0 and 81.4% for 50), and the open pore percentage, which, in both cases, turned out to be up to 98.0% and 98.8%, respectively. According to the literature, maximizing porosity is crucial for enhancing the thermal insulation of open-cell foams, as it helps achieve the low thermal conductivity of air, which is 0.026 W/(m·K) under standard conditions [42]. In order to complete the intrinsic properties of these foams, BET analysis was also performed to establish the surface area of the monoliths. These measurements were very hard to obtain because around 25% of the material evaporates during the vacuum phase. The measured isotherms allowed us to determine no nanoporosity and estimate surface areas lower than 1 m^2^/g. A comparison of these foams with physically blown furanic foams [43,44] indicates that the mechanically blown SPI-protein foams result in higher densities and lower porosity but also fewer orthotropic cells. In terms of cell dimension, open porosity, and surface area, the results are comparable to those of tannin–furanic foams.

### 3.2. B-Chemical Analysis

**Figure 3:**
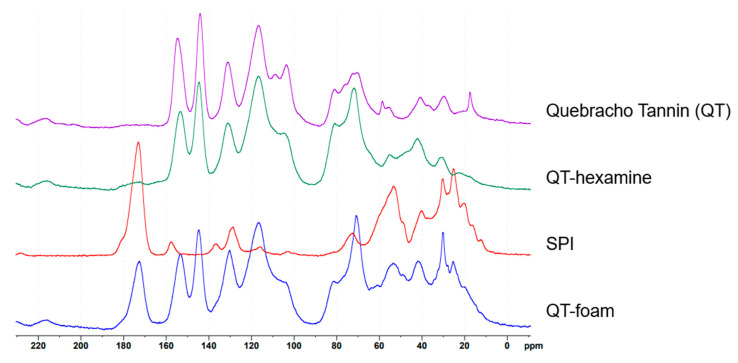
Spectra for quebracho foam spectra from top to bottom: tannin alone, tannin reacted with hexamine, SPI, and the final foam polymer.

Solid-state ^13^C-NMR analysis was performed on various components and their reaction products of the 100% and 0% chestnut foam formulations. The spectra were interpreted by literature comparison and simulating potential chemical structures using the NMR prediction tool (nmrdb.org) [45]. Figure 3 displays the spectra obtained for the different components and reaction products of quebracho tannin.

**Figure 4:**
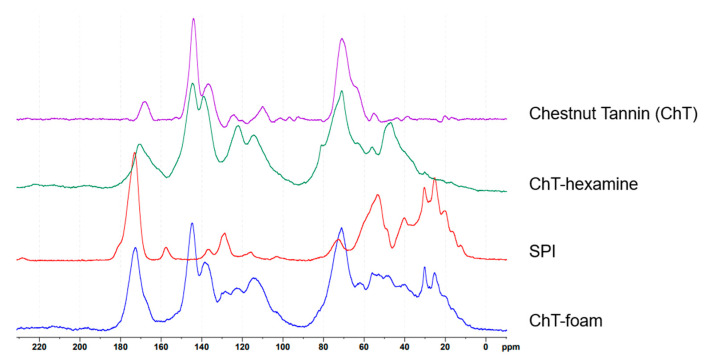
Spectra for chestnut tannin foam analysis.

Comparing the spectra of quebracho tannin with that of polymerized quebracho with hexamine, it is possible to highlight some differences that suggest the way hexamine covalently combines with the extract. In the region between 125 and 96 ppm, there is a clear increase of the band at 117 and a decrease of that at 104 ppm. In this area resonate the -OH-free aromatic carbons of the A ring of the flavonoid. In particular, the ones keeping a simple hydrogen resonate at 104 ppm, while the substituted ones (including the interflavonoid connections) resonate at around 117 ppm. This means that the hexamine moieties can occupy the free position of the A ring during the curing by forming methylene bridges. The crosslinking behavior of hexamine (hexamethylene tetramine) is complicated since this three-dimensional structure can unfold to different degrees and eventually also produce moieties, which could all contribute to crosslinking. The degree of unfolding of hexamine can be observed with the distribution of CH_2_ signals in the area around 70 (more folded) and 40 ppm (more unfolded) [46]. The slight increased signal at 81 in the reacted spectra of QT-hexamine can also be explained by cyclic ether-type formations from hexamine or formed -N = CH-N(CH_2_)-CH_2_OCH_3_ groups [47]. In the region around 71 ppm, the foam and QT-hexamine spectra show a signal increase compared to tannin alone, with a higher increase in QT-foam than in QT-hexamine. This may be due to the presence of cyclic hexamine structures or the presence of -NH-CH_2_-O- groups [47]. The second could therefore suggest possible reactions of the hexamine part on the hydroxyl group of the phenolic ring, while the 117 ppm increase indicates crosslinking at the unoccupied A ring position. In QT-hexamine, the 117 ppm peak is more prominent, while in the foam, the 71 ppm peak is higher, suggesting that in the presence of SPI, hexamine may form more cyclic structures or promote reactions at the –OH group. However, these interpretations are tentative and require further analysis. The hexamine unfolding process was similarly observed with tannin reacted in an alkaline environment [46,48]. The spectrum of the Quebracho foams is well summarized by the overlapping of the spectra of tannin–hexamine polymer and SPI. This means that no new signals are formed in the presence of SPI; however, considering the complexity of the different groups in the proteins, the presence of these signals would be hard to detect. The spectra of the chestnut components and reactants are reported in Figure 4.

**Figure 5:**
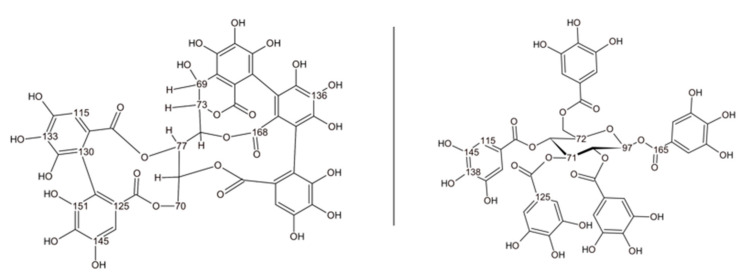
Chemical structure of vescalagin (**left**) [53] and pentagalloylglucose (**right**) as representative structures of chestnut tannin.

Chestnut tannin is a complex mixture of polyphenolic compounds, predominantly composed of ellagic acid derivatives, such as castalagin and vescalagin, followed by gallic acid and its derivatives, also referred to as gallotannins [49,50,51,52]. Figure 5 illustrates the structural differences between vescalagin and pentagalloyl glucose, serving as representative compounds for understanding the reaction mechanisms involved.

**Figure 6:**
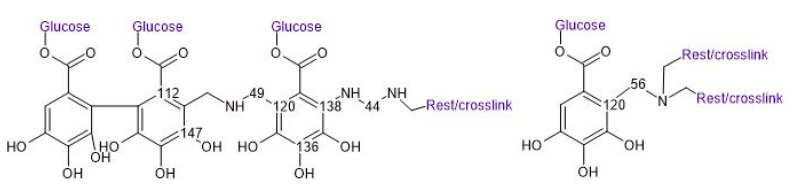
Hypothetical crosslinking reaction of hexamine and units of ellagitannin/gallotannin compounds.

Based on these chemical model structures, several observations about the spectra can be drawn. Significant changes in the 30–60 ppm region of the spectra for reacted tannin, compared to the tannin extract, indicate the formation of new bonds. These peaks are linked to the reaction of hexamine with tannin as already described for the quebracho polymers. It has been shown that, under acidic conditions, hexamine does not merely degrade to formaldehyde; instead, it can form complex intermediates that can further react with the tannin [47,54] and are also found in the reacted chestnut spectra. This explains the broad range of signals observed, reflecting the complexity of the reaction products. The peak at 48 ppm suggests -CH_2_-NH-CH_2_- groups attaching to previously free ortho positions on the phenolic ring, acting as a crosslinker for the phenolic units. The peak at 56 ppm further indicates the development of a more branched network when hexamine crosslinks with chestnut tannin (Figure 6). Comparing ChT reacted with hexamine to ChT-foam with SPI, the 49 ppm signal decreases in relation to the signal at 56 ppm, indicating that SPI reduces the polymer network’s linearity and increases branching. In the ChT reacted with hexamine, a small peak at 81 ppm, likely from unreacted hexamine or specific hexamine-related structures [47], is significantly reduced in the foam spectra, but the pattern for the partly unfolded hexamine, visible in the slightly lower region, remains similar. The 130–155 ppm region corresponds to the aromatic carbons attached to the -OH group, but their resonance differs between the reacted spectra and the extract’s. In the case of gallic acid, the two carbons in meta position resonate at 145 ppm, while that in para resonates at 138 ppm. When chestnut polymerizes with hexamine, the signal at 137–139 ppm becomes higher due to the direct substitution of the ortho position with nitrogen from hexamine. A shift to lower fields occurs also in the signal of the carboxyl carbon, which confirms a new position where hexamine creates a covalent connection with the phenolic ring. When hexamine crosslinks via the CH_2_-R group to the free ortho position of the phenolic ring, the increase in the signal at 122 ppm for the reacted tannin, along with the associated shift at 48 ppm, can be explained. The overall differences between the ChT-hexamine and the ChT-foam spectra can mainly be explained by overlapping the ChT-hexamine spectra with those of SPI. The spectral analysis allowed for the detection of covalent bond formation by hexamine with the tannin, which led to a more branched rather than linear structure in the presence of SPI. However, detecting covalent bonds involving SPI is challenging, as many signals in the 30 to 55 ppm region overlap with hexamine-derived formations. Based on the spectral analysis, we can suggest the crosslinking reaction mechanism based on hexamine with ellagi- and gallotannins as depicted in Figure 6.

### 3.3. C-Physical Properties

**Figure 7:**
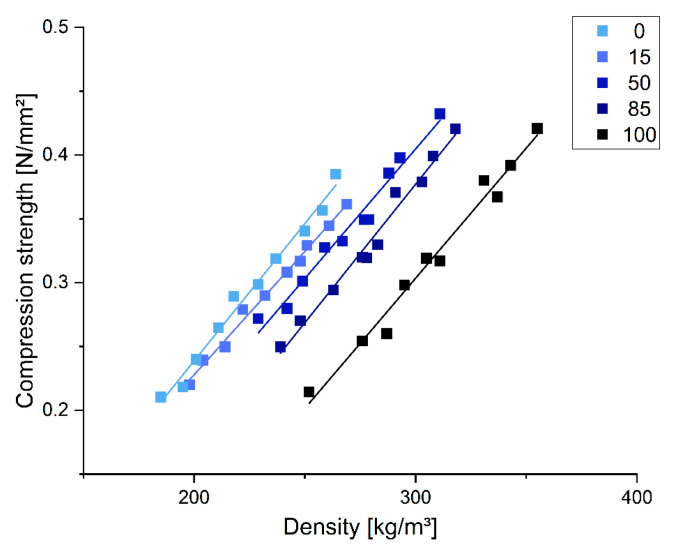
Compression resistance of different foam formulations according to sample’s density.

Figure 7 shows the compression test results, showing the relationship between the specimen’s density and the recorded value at which a break occurred in the material.

Both the density and type of tannin used have a significant impact on the compression strength of the foams, also confirmed by multiple regression analysis (*p* < 0.001; R^2^ 0.884). When comparing foams with similar densities, it was observed that an increase in chestnut tannin content led to a decrease in compression strength. Both pure chestnut and quebracho foams show lower compression strength than tannin–furanic and tannin–albumin foams produced through a similar method [18,30]. For instance, chestnut tannin–furanic foams with a density of 258 kg/m^3^ showed a compression strength of 1.02 N/mm^2^, while quebracho tannin–furanic foams at 244 kg/m^3^ had a compression strength of 1.21 N/mm^2^. These results align with these previous findings that chestnut furanic foams tend to have lower compression strength compared to their quebracho counterparts. In the case of hydrolysable tannin foams, both their densities and compression strength are comparable to those of NIPU foams reported in the literature, which range from 310 to 350 kg/m^3^ in density and 0.32 to 0.91 N/mm^2^ in compression strength [19]. The foams presented here have a higher tannin content relative to the cross-linking agent when compared to NIPU foams in the literature where the hexamine and glutaraldehyde ratio was found to be the main factor influencing compression strength, ahead of density [19]. The results of the thermal conductivity measurements according to the tested density are shown in Table 3.

As known from literature, thermal conductivity in tannin foams is primarily influenced by material density and porosity, and this trend was confirmed also in our tests [42]. Upscaling the foams increased density in those with 50% to 100% chestnut tannin. While the quebracho-only foam (0%) had lower thermal conductivity than the 15% chestnut foam at similar density, no clear effect of chestnut content on thermal conductivity was observed. Instead, material density was the dominant variable. Compared to foams made with albumin using a similar method, the foams here generally showed lower thermal conductivity at comparable densities (231 to 326 kg/m^3^ and 67.7 to 80.5 mW/(m·K) [30]. In addition, the foams produced in this study generally have a higher thermal conductivity than tannin–furanic foams produced by mechanical agitation, which at comparable densities (260 to 300 kg/m^3^) have thermal conductivities of 52 to 55 mW/(m·K) [18].

**Figure 8:**
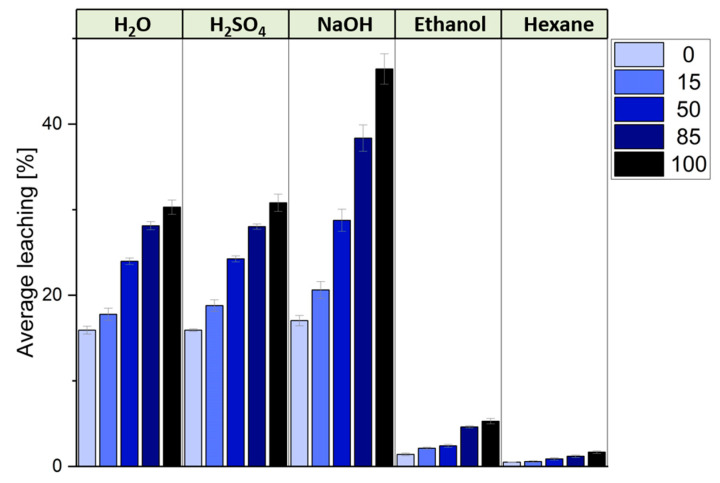
Leaching resistance of the different foam formulations in various environments.

Leaching tests were performed in various environments to assess the chemical resistance and estimate the crosslinking degree of the produced foam materials. Figure 8 illustrates the average leaching resistance of tannin-based foams when tested across different solvents.

Since approximately 20% by weight of the non-water components in the foam formulation—ethylene glycol, glycerol, Tween 80, and sulfuric acid—are expected to remain unreacted in the polymer and are more soluble, leaching rates higher than this value clearly indicate the presence of unreacted tannin extract. In water, the leaching ranged from 16% to 30%, being the highest for the pure chestnut formulation. As the proportion of quebracho tannin increased, the leaching decreased. The 16% leaching observed in the quebracho foam aligns well with existing studies, which report approximately 14% leaching for a quebracho furanic formulation, even though the presented formulations contain a lower percentage of crosslinker [18]. The higher leaching in chestnut foams indicates unreacted tannin fractions. When tested in an acidic environment at pH 2, the leaching behavior was similar to that in neutral water, which could be expected since the foams were produced under acidic conditions. Conversely, in an alkaline environment (pH 13), the leaching patterns differed. While the leaching rates for the quebracho foam remained similar to those in water, the chestnut tannin foams exhibited significantly higher leaching, indicating that chestnut tannin polymers are cleaved in these conditions and hence less resistant to alkaline conditions than those made with quebracho tannin. As expected, the foams also demonstrated greater overall resistance to leaching in less polar solvents such as ethanol and hexane, with leaching rates ranging from 1.4% to 5.3%. Also, for these solvents, the leaching was higher with increasing chestnut content. Overall, the quebracho foams showed strong resistance to leaching in various environments, including water, acid, base, ethanol, and hexane. In contrast, chestnut foams exhibited higher leaching rates, particularly in alkaline conditions, indicating lower chemical resistance and the appearance of an unreacted fraction coming from the tannin extract.

**Figure 9:**
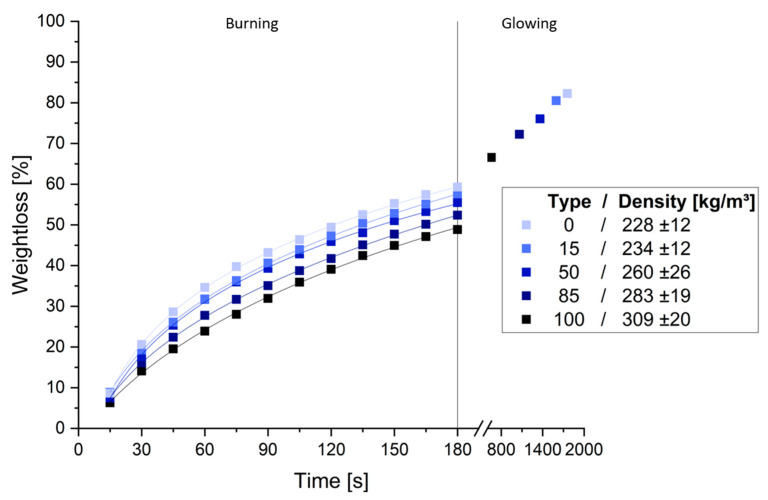
Weight loss during fire testing with 180 s of direct flame exposure, followed by a glowing phase until weight stabilization, for various foam formulations.

The average weight loss of different tannin-based foams during 180 s of direct flame exposure and the final stabilized weight loss are shown in Figure 9.

All foams exhibited a rapid initial mass loss, followed by a slower, more linear decline. After the flame was extinguished, the foams stopped burning but continued to glow for 8 to 26 min until weight stabilization. Foams with lower weight loss during the initial burning phase consequently had shorter glowing times. Weight loss during the burning phase ranged from 49% to 59%, with quebracho foams showing the highest loss and chestnut foam the lowest. Final weight loss ranged from 67% to 82%, consistent with the burning phase trends. Multiple regression showed no significant effect of tannin type on final weight loss, but density was a significant predictor (*p* < 0.001, R^2^ 0.919). When compared to lightweight tannin–furanic foams from the literature, the foams in this study exhibit a comparable weight loss after 180 s of flame exposure but differ significantly with a much longer glowing phase and greater additional weight loss [38]. Given the higher density of the tannin–protein foams, the tannin–furanic foams appear to offer better fire resistance. Overall, the foams were self-extinguishing and non-flammable but showed a relatively long glowing behavior.

## 4. Conclusions

This study demonstrated the feasibility of using chestnut tannin in hexamine-protein-based foam formulations, highlighting its potential as a substitute for quebracho tannin. The inclusion of soy protein isolate (SPI) allows for the effective use of low amounts of hexamine, further enabling the formulation of foams with up to 100% chestnut tannin. However, while chestnut tannin can be utilized, quebracho tannin proves easier to process due to its higher reactivity, leading to foams with lighter densities and significantly better mechanical properties at comparable densities. The morphological analysis revealed that foams with higher quebracho content exhibit smaller cells with thinner walls and more interconnections, whereas increasing the proportion of chestnut tannin results in larger, thicker, and less interconnected cells. The fire resistance tests showed that higher-density foams provided better fire resistance, with no significant impact from the type of tannin used. The study also found that formulations with higher chestnut tannin content are more sensitive to leaching, particularly in water and an alkaline environment. Additionally, higher chestnut content increased thermal conductivity, primarily due to higher density rather than the tannin type. The skeletal density of the foams remained consistent across formulations at 1.45 g/cm^3^, with porosity being very similar, characterized by approximately 98% open cells. The ^13^C-NMR analysis revealed that hexamine covalently bonded with quebracho and chestnut tannin. In the presence of SPI, the chestnut polymers seem to become more branched compared to hexamine alone; however, the covalent bonding with proteins was not directly detected. Overall, the study showed that chestnut tannin can replace quebracho tannin in hexamine–SPI foam formulations, but blending condensed and hydrolysable tannins may offer a better compromise for low density and mechanical strength, opening the field for exploring other feedstocks composed of mixtures of these two for tannin foam production.

## Figures and Tables

**Table 1:** General formulation of characterized tannin foams expressed in percentage by weight (wt%).

Tannin [%]	SPI [%]	Hexamine [%]	Ethylene Glycol [%]	Glycerol [%]	H_2_O [%]	Tween [%]	H_2_SO_4_ [%]
43.78	12.26	4.38	6.13	2.63	23.35	4.67	2.8

**Table 2:** Results of cell size measurements.

Name	Density [kg/m^3^]	Calc. Cell Diameter [µm]	Orthotropicity [%]
0	225 (26)	158 (49)	1.21 (0.13)
15	234 (23)	178 (57)	1.20 (0.10)
50	269 (24)	217 (77)	1.21 (0.12)
85	281 (24)	241 (77)	1.23 (0.07)
100	309 (31)	283 (109)	1.23 (0.14)

**Table 3:** Thermal conductivity measurements of upscaled formulations.

Name	Density [kg/m^3^]	Thermal Conductivity [mW/(m·K)]
0	230	53.7 (1.9)
15	235	62.6 (1.0)
50	310	72.6 (1.6)
85	350	76.8 (1.4)
100	570	113.2 (2.4)

## Data Availability

The data are available upon request.
